# Insights into the effects of different drying methods on protein oxidation and degradation characteristics of golden pompano (*Trachinotus ovatus*)

**DOI:** 10.3389/fnut.2022.1063836

**Published:** 2022-11-24

**Authors:** Peng Chen, Yingjie Qiu, Shengjun Chen, Yongqiang Zhao, Yanyan Wu, Yueqi Wang

**Affiliations:** ^1^College of Food Science and Technology, Guangdong Ocean University, Zhanjiang, China; ^2^Key Laboratory of Aquatic Product Processing, Ministry of Agriculture and Rural Affairs, National R&D Center for Aquatic Product Processing, South China Sea Fisheries Research Institute, Chinese Academy of Fishery Sciences, Guangzhou, China; ^3^College of Food Science and Engineering, Ocean University of China, Qingdao, China; ^4^Collaborative Innovation Center of Seafood Deep Processing, Dalian Polytechnic University, Dalian, China

**Keywords:** golden pompano, hot air drying, heat pump drying, myofibrillar proteins, oxidative properties

## Abstract

The quality of dried fish products differs based on the drying method employed owing to the different drying principles, with changes in protein affecting the quality of these products. Therefore, we investigated the differences in golden pompano (*Trachinotus ovatus*) fish tissue structure and protein physicochemical properties under different drying methods. Freeze drying (FD) induced less tissue damage, leaving more intact myofibrils, than that of hot air drying (HAD) and heat pump drying (HPD). The structural stability of myofibrillar protein was retained to a greater extent after FD, while myoglobin oxidation was lower, and fish meat color was well maintained. Our findings not only elucidated the effects of several drying methods on the physicochemical properties of fish protein, but also determined the mechanism underlying quality changes observed during the drying process. This provides a theoretical reference for the study of dried fish filet processing.

## Introduction

*Trachinotus ovatus*, commonly known as golden pompano, widely distributed throughout the Pacific and Indian Ocean, is an economically important mariculture fish species for many Southeast Asian countries (1). Owing to its bright body color and delicious meat, golden pompano is favored by the majority of consumers. As a result, the yield of golden pomfret has increased along with advances in processing technology. The processing of fish into semi-dried products inactivates enzymes and inhibits the growth of microorganisms, improving fish freshness; however, it may also impart a unique taste to the product (2). At present, the most common means of drying employed during the processing of aquatic products are hot air drying (HAD), heat pump drying (HPD), and freeze drying (FD). These drying methods differ in their principles of water loss, which also gives rise to differences in the quality of their respective dried products (3). Traditional HAD is a method of drying the food surface using hot air as the drying medium, with the advantages of low cost and easy operation. However, high temperature at the food surface may cause surface sclerosis, preventing complete diffusion of the internal moisture, which leads to uneven drying inside and outside. HPD utilizes heat drawn from low-temperature heat sources and converts the heat into high-grade thermal energy to dry food products. It is more gentle than HAD, with the advantages of uniform drying and ease of control. Nevertheless, disadvantages include a long drying time and high equipment maintenance requirements. The introduction of vacuum FD technology has allowed for improvement in the quality of aquatic products (4). Vacuum FD is achieved by freezing moisture inside the food and then sublimating the ice under vacuum to dryness. The products obtained *via* vacuum FD exhibit less structural damage and a lower loss of nutrients, which has led to its wide utilization throughout the food industry (5).

Nevertheless, aquatic products are prone to deterioration during the drying process, mainly as a result of the Maillard reaction as well as protein and lipid oxidation. Yin et al. (6) compared the effects of HAD, vacuum HAD, microwave drying, and vacuum FD on the taste-determining components of scallop endocardium and reported the highest free amino acid and fresh nucleotide contents after vacuum FD. Qiu et al. (2) explored differences in the sensory qualities and flavor of golden pompano subjected to different drying conditions, observing considerable flavor differences between drying temperatures and superior sensory characteristics at a drying temperature of 40°C. Whereas these studies demonstrated that different drying treatments can affect product quality to varying degrees, existing research has focused mainly on flavor and lipid oxidation, whereas the changes in protein, as a source of free amino acids, have rarely been studied in the context of drying (7).

Protein oxidative degradation is directly or indirectly related to environmental factors, such as temperature, humidity, and oxygen during the drying process. Myofibrillar protein, which accounts for 50–70% of the total protein in muscle, has distinct structural and functional properties (8). The stability of muscle tissue structure is dependent on the protein spatial meshwork, and chemical interactions are critical for maintaining the meshwork (9). Myofibrillar proteins undergo oxidative denaturation during processing, manifested by protein backbone breakage, protein cross-linking, and amino acid side chain modification. Conformational changes in myofibrillar protein are an important indicator of fish product quality (10). A moderate oxidation of proteins helps in the formation of some taste substances, whereas excessive oxidation may lead to off-flavors, which potentially affect the overall flavor and color of the product. Therefore, we aimed to investigate differences in fish tissue and changes in protein under different drying methods. To this end, we characterized the structural properties and microstructure of golden pompano fish through an analysis of the oxidative degradation of myogenic fibrin and myoglobin and changes in intermolecular interaction forces, in an attempt to determine the mechanism underlying protein changes influencing fish quality. The results can elucidate the effects of different drying methods on product quality and provide a theoretical basis for improving the quality of dried marine fish products.

## Materials and methods

### Sample preparation

Fresh adult golden pompano (weight, 500 ± 50 g; length, 28 ± 2 cm) were purchased from a fish farm in Guangdong Province, China. The samples were transported to the laboratory at 4°C within 2 h of purchasing. The viscera and gills were removed from the fish, which were subsequently washed with water to remove blood and dirt. Subsequently, 2.5 × 2 × 1 cm filets were cut from the same location on the back of each fish for drying.

### Drying treatment

For HAD, fish filets were placed in a HAD oven (DHG-9145A, Yihen Co., Ltd., Shanghai, China) equipped with a blower. The temperature was set to 60^°^C for constant-temperature drying until moisture was reduced to 20 ± 2%, at which point samples were removed from the oven.

For HPD, samples were placed in a heat pump dehumidification and drying oven (RCX-1500-1540, Xiangao Refrigeration Equipment Co., Ltd., Foshan, China) until moisture was reduced to 20 ± 2%.

For FD, filets were pre-frozen at 80 ^°^C for 4 h and placed in an FD oven (Alpha1-4, Christ Co., Ltd., Germany) with a cold trap temperature of –40 ^°^C and a vacuum of 20 Pa until moisture reached 20 ± 2%.

### Texture profile analysis

The texture profile of the fish filets was analyzed as per the method of Ben Atitallah et al. (11), with slight modifications, using a texture analyzer (TA Plus, Lloyd Instruments, Bognor Regis, UK). A 50 mm diameter cylindrical P25 probe was used with the following parameters: 60% compression deformation, 5 g trigger force, 3 mm/s pre-test speed, 1 mm/s test speed, 1 mm/s post-test speed, 5 s test interval, and 50% compression ratio.

### Scanning electron microscopy

The filets were fixed in 2.5% glutaraldehyde overnight, sequentially dehydrated with a gradient of 30, 50, 70, and 90% ethanol; freeze-dried for 48 h, and photographed under a scanning electron microscope (Image Scanner III, General Electric Company, Schenectady, NY, USA) after gold spraying by ion sputtering (12).

### Extraction of myofibrillar protein

We used the method of Liu et al. (13), with slight modifications. First, 2.00 g of fish was added in a 50 mL centrifuge tube, followed by the addition of 20 mL phosphate buffer solution (PBS) A (3.5 mmol/L NaH_2_PO4, 15.6 mmol/L Na_2_HPO4, pH 7.5). After homogenizing and mixing in an ice bath (10,000 *g*, 2 min), extraction was performed at 4 °C for 30 min, followed by centrifugation (4°C, 5,000 *g*, 15 min) and addition of 20 mL PBS A to the precipitate. After extractions, the precipitate of PBS A was mixed with 20 mL PBS B (3.5 mmol/L NaH_2_PO_4_, 15.6 mmol/L Na_2_HPO_4_, 0.5 mmol/L NaCl, pH 7.5), homogenized and mixed in an ice bath. The mixture was centrifuged after 30 min of extraction, whereafter 20 mL PBS B was added to the precipitate, and the operation was repeated. The supernatant obtained was combined to yield the myofibrillar protein.

### Sodium dodecyl sulfate polyacrylamide gel electropheresis

Based on the method of Li et al. (14), the myofibrillar protein solution was diluted to 1.00 mg/mL and mixed with 2 × Sodium dodecyl sulfate polyacrylamide gel electropheresis (SDS-PAGE) loading buffer in equal volume. The samples were incubated in a water bath at 100°C for 5 min and then centrifuged at 12,000 rpm for 5 min, and the supernatant collected. Electrophoresis of the samples (10 μL loading volume) was carried out in 1 × running buffer using an initial constant voltage of 80 V to concentrate the sample, whereafter the voltage was switched to 120 V until the bromophenol blue indicator reached the bottom edge, and electrophoresis was stopped. The protein bands were stained with Coomassie brilliant blue R250 for 45 min, decolorized with water until the background was colorless, and then finally scanned and analyzed.

### Determination of total sulfhydryl and carbonyl content

The total sulfhydryl content and carbonyl content of myogenic fibronectin was determined using the Total Sulfhydryl Content Determination Kit (A063-2-1) and the Carbonyl Content Determination Kit (A087-1), respectively.

### Determination of surface hydrophobicity

Surface hydrophobicity was determined based on the method of Chelh et al. (15), with appropriate modifications. The above myofibrillar protein was diluted to 2 mg/mL with 20 mM phosphate buffer (pH 6.0), after which 1 mL of the diluted solution was mixed well with 100 μL of 1 mg/mL bromophenol blue. Phosphate buffer was used as blank control. Samples were vortex-shocked for 10 min, freeze-centrifuged (3,000 rpm, 15 min), and the resulting supernatant diluted 50-fold with phosphate buffer. The absorbance values at 595 nm were measured in parallel for each sample. Surface hydrophobicity was determined as per the following equation:


(1) $$\text{Bromophenol}\,\text{blue}\,\text{binding}\,\mathrm{amount}=100\times \frac{\left({\mathrm{Control}}_{\text{A}}\;-\;{\text{Sample}}_{\text{A}}\right)}{{\text{Control}}_{\text{A}}}$$

where Control_*A*_ represents the absorbance value of the control group, and Sample_*A*_ represents that of the sample group.

### Fourier transform infrared spectroscopy

Referring to the method of Li et al. (16), the myofibrillar protein solution was adjusted to the same protein concentration (1 mg/mL), frozen at –80°C for > 4 h, and placed in a vacuum freeze dryer until completely dry. The lyophilized myofibrillar protein samples were mixed with anhydrous potassium bromide at a ratio of 1:100 (m/v) thorough grinding, pressed into slices, and analyzed on a Fourier infrared spectrometer (IRAffinity-1, Shimadzu, Japan). The infrared spectra were measured at a resolution of 4 cm^–1^, with 24 repeated scans in the range of 4,000–400 cm^–1^. The results were fitted with a Gaussian fit using the Peak fit 4.21 software to determine the secondary protein structure.

### Measurement of endogenous fluorescence intensity

We employed the method described by Lina et al. (17), with slight modifications. The myofibrillar protein solution was diluted to 0.05 mg/mL with 0.6 M NaCl solution, and endogenous fluorescence intensity was measured using a fluorescence spectrophotometer (UV2550, Shimadzu, Japan). An excitation wavelength of 295 nm and an emission wavelength scan range of 200–500 nm was used at a scan speed of 1,000 nm/min and an excitation and emission slit width of 10 nm.

### Chemical interactions

Referring to the method of Tan et al. (18), we combined 3 g of sample with 30 mL of S1 (0.6 M NaCl) and homogenized the sample in an ice bath at 1,000 rpm for 2 min, before allowing it to rest for 1 h. After centrifugation at 10,000 rpm for 25 min, the supernatant was stored at 4 °C. After centrifuging again to obtain the precipitate, 30 mL of S2 (0.6 M NaCl + 1.5 M urea) was added to the precipitate, and the above steps were repeated. The precipitate was then mixed with 30 mL of S3 (0.6 M NaCl + 8 M urea), the above steps were again repeated, whereafter 30 mL of S4 (0.6 M NaCl + 8 M urea + 0.5 M β-mercaptoethanol) was added to the precipitate. After repeating the above steps, 2 mL of 1 M NaOH was added to the obtained precipitate for preservation. All the supernatants obtained were added to the same volume of 20% trichloroacetic acid solution, centrifuged at 5,500 rpm for 15 min, and the precipitates collected. Again, 2 mL of 1 M NaOH was added to the precipitates for preservation, whereafter their protein content was determined using the Lowry method. All of the above processes were carried out at 4°C. Relative to the total protein content, S1 protein represents ionic bonds, S2 protein represents hydrogen bonds, S3 protein represents hydrophobic interactions, and S4 protein represents disulfide bonds. The S4 protein content relative to total protein content precipitated after centrifugation represents non-disulfide covalent bonds.

### Measurement of myoglobin content

Base on the method of Krzywicki (19). Meat samples (5 g) were combined with 5 × volume of 0.04 M sodium phosphate buffer (pH 6.8) and homogenized for 25 s in a homogenizer at 10,000 rpm. The mixture was then placed in a homogenizer at 4°C for about 1 h for full extraction, followed by centrifugation at 4,500 rpm for 20 min at 4°C. The supernatant was filtered, and absorbance values were measured at 525, 545, 565, and 572 nm using a spectrophotometer. The content of different forms of myoglobin content was determined as follows:


(2) Myoglobin(%)=(0.369⁢A1+1.140⁢A2-0.941⁢A3+0.015)×100

(3) Oxygenatedmyoglobin(%)=(0.882⁢A1-1.267⁢A2+0.809⁢A3-0.361)×100

(4) Highironmyoglobin(%)=(-2.514⁢A1+0.777⁢A2+0.800⁢A3+1.098)×100

where A1, A2, and A3 are the absorbance ratios A572/A525, A565/A525, A545/A525, respectively.

### Statistical analysis

All analytical tests were repeated three times, and data were analyzed using SPSS 23.0 (SPSS Inc., Chicago, IL, USA). Duncan’s multiple comparison was used for the analysis of variance. Statistical significance was set at *p* < 0.05. Texture heat maps were drawn using the TBtools software (v1.09), and column charts using Origin 2021.

## Results and discussion

### Textural profile of fish filets

**FIGURE 1:**
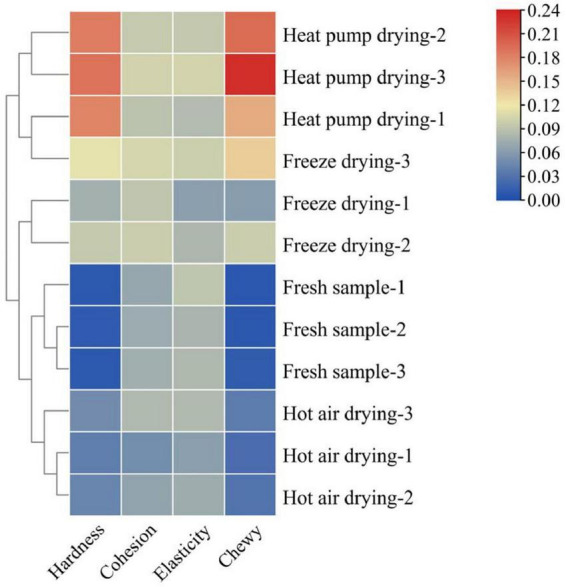
Effects of different drying methods on the texture of fish filets. Color indicates the value: red indicates the higher value, blue indicates the lower value.

Texture characteristics are important sensory indicators for evaluating dried aquatic products, and changes in the texture of fish during drying is an important contributing factor to the decline in quality (20). Figure 1 shows the changes in fish filet texture under several drying treatments. The hardness and chewiness of the filets showed a significant increase as a result of different drying treatments, being significantly higher in the HPD group than in the other groups. No significant difference was observed in cohesion among the groups (*p* > 0.05), while the elasticity of fish filets of the HAD group was lower than that in the other groups. Water content is an important factor affecting fish filet texture. As a result of the heating of the filet surface during drying, water in the fish flesh is gradually lost, leading to an increase in hardness (21).

Protein structural changes also affect the texture of fish to a certain extent. Wu et al. (22) reported that myofibrillar protein was significantly affected under different heating conditions, especially under conditions of a high-temperature environment. Aggregation and cross-linking of proteins was observed, while myofibrils contracted longitudinally and intermolecular gaps increased, which in turn changed the texture of the product. This may explain the lower elasticity of fish meat in the HAD group. The higher the drying temperature and the longer the drying time, the higher the degree of protein denaturation, the more compact the product structure, and the greater the hardness and chewiness. In general, products with moderate hardness and chewiness are acceptable to most consumers, whereas products that are too hard or soft will affect taste. Taken together, the FD product was moderately hard, elastic, and chewy, with good textural properties.

### Changes in microstructure of fish filet tissue

**FIGURE 2:**
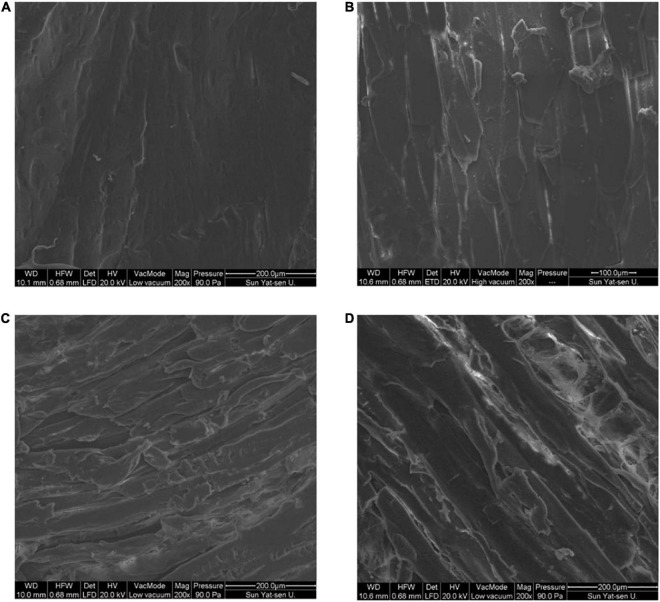
Effect of different drying methods on the tissue microstructure of fish filets. **(A)** Fresh sample; **(B)** hot air dried sample; **(C)** heat pump dried sample; **(D)** freeze dried sample.

After drying, the moisture content of fish was lower, and the internal tissue microstructure was changed. The rehydration and water-holding capacities of dried products are closely related to their microstructure (23). The microstructure of the fish flesh under different drying methods is shown in Figure 2. The muscle tissue fibers of fresh fish meat were smooth and flat, with a tight and orderly arrangement. Following HAD, muscle fibers exhibited a low amount of breakage. This could be due to the fact that high temperatures destroy the muscle bundle membrane structure, altering fibers, while the loss of water provides space for fiber contraction, causing the muscle fibers to contract continuously and the muscle tissue to become denser and harder (24). Following HPD, the surface of the fish muscle fibers was rough and shrunk significantly, with a more disorganized arrangement and looser structure compared with that subjected to HAD. There were also obvious fractures. This may have been due to the relatively low drying temperature of the heat pump and long water evaporation time, leading to less muscle fibers contraction. During FD, the water changes from liquid to solid ice crystals, and the dehydration process involves direct sublimation from the solid to gaseous state. Thus, the gap in fish myogenic fibers becomes larger after FD, and honeycomb-like holes appear, which is also explains why the volume of freeze-dried fish filets expands slightly and the rehydration capacity is stronger (25, 26). After drying, the fibrous structure was intact, without obvious shrinkage, and a smooth surface was observed. Filamentous proteins could be observed in pores, indicating that FD induced the least damage to fish tissue when compared with the other drying methods.

### Protein degradation

**FIGURE 3:**
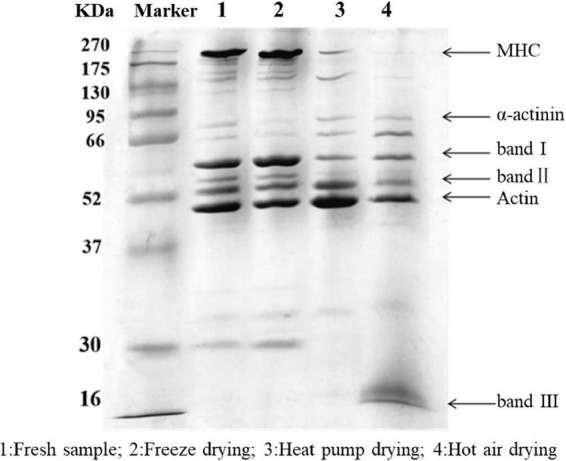
SDS-PAGE of golden pompano myofibrillar protein. Lane 1: Fresh sample; 2: Freeze dried sample; 3: Heat pump dried sample; 4: Hot air dried sample.

The results of SDS-PAGE are shown in Figure 3. Overall, the protein bands of fresh fish and FD samples were more dense and greater in number, while those for HAD and HPD fish were less dense. Myosin heavy chain (MHC) with actin was clearly present in fresh and FD fish. α-Actin bands in FD fish showed a slight reduction whereas small-molecular weight protein bands were more dense. This indicates that FD largely prevented myofibrillar protein degradation, although slight protein denaturation did occur, which may be related to the freezing process (27).

After HPD, the density of MHC was significantly reduced, with protein band I becoming progressively lighter, and actin and protein band II denser than in the other groups. These results indicate that MHC was degraded into small molecular weight proteins between 37 and 95 kDa, which is similar to the results of Deng et al. (28). After HAD, the MHC band almost completely disappeared, and there was a significant decrease in the intensity of band II compared with that of the actin band, and a new band III appeared at 16 kDa. Cai et al. (29) reported that myosin is highly sensitive to oxidation due to the presence of cysteine residues. When the temperature reaches 60°C, myosin, actin, and myosin light chains are strongly degraded, which may be due to the generation of free radicals. Protein polymerization occurs as a result of cross-linking between molecules, with various oxidation products being generated at the same time (30). High temperature during HAD might have caused some proteins to polymerize and form large molecules that could not enter the separation gel. Meanwhile, high temperature reduced the thermal stability of proteins such as myosin and actin, which were degraded into smaller protein molecules. In conclusion, myofibrillar proteins in the fish filets were subjected to various degrees of degradation under the action of free radicals generated during drying, high temperatures, and freezing denaturation.

### Oxidative degradation of myofibrillar proteins

**FIGURE 4:**
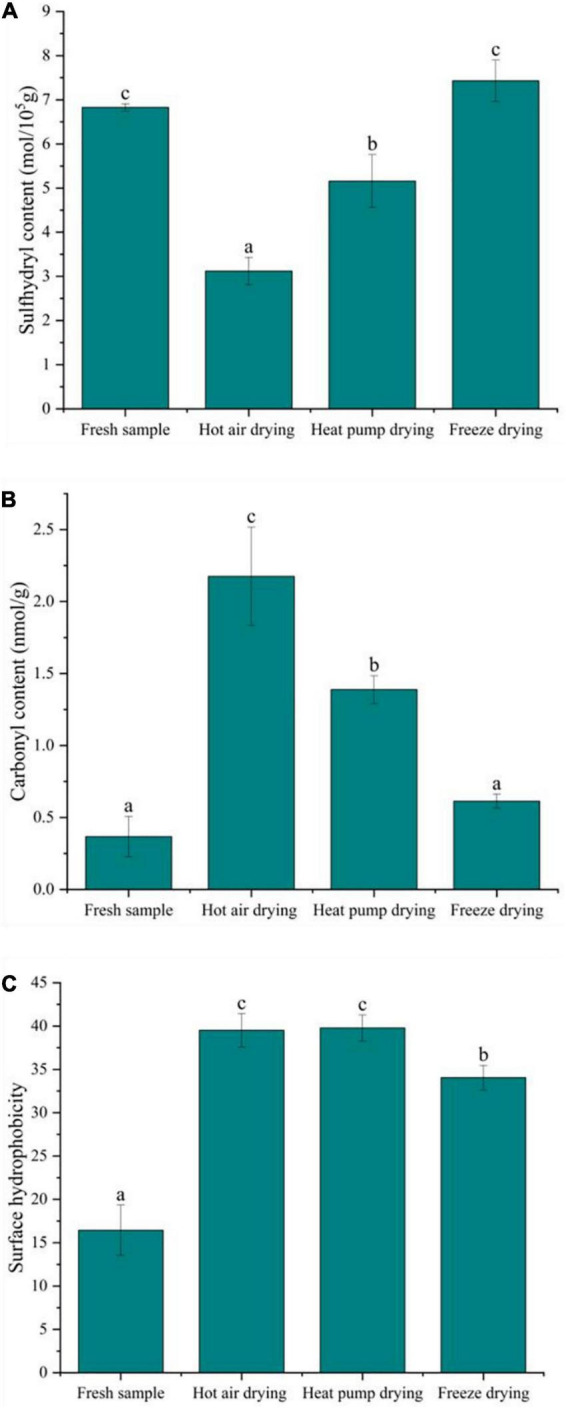
Effect of different drying methods on the oxidative degradation properties of myofibrillar protein. **(A)** Sulfhydryl content; **(B)** carbonyl content; **(C)** surface hydrophobicity. Values represent the mean ± SD (*n* = 3). Lower case letters indicate significance at *p* < 0.05.

The formation of sulfhydryl groups in proteins results from the single-electron oxidation of cysteine by free radicals. Cysteine is susceptible to attack by reactive oxygen species (ROS), which causes changes in sulfhydryl content; thus, sulfhydryl content reflects the degree of protein oxidation. As shown in Figure 4A, the total sulfhydryl content of myofibrillar protein after FD was basically the same as that of fresh fish, confirming that FD had no significant effect on sulfhydryl content (*p* > 0.05). In contrast, there was a significant decrease in sulfhydryl content after HAD and HPD (*p* < 0.05), with decreases of 54.3 and 24.5% compared to fresh fish, respectively. During HAD and HPD, the high-temperature environment promotes the formation of disulfide bonds and protein aggregates (31). Cheng et al. (32) previously reported that heat stress affects mitochondrial function, causing free radical accumulation, leading to structural changes in myofibrillar proteins. Further, FD involves a shorter drying time compared to the other two drying methods, which prevents further oxidation of proteins to a certain extent.

The formation of carbonyl groups in fish myofibrillar protein is an important indicator of protein oxidation and occurs in four ways: the side chains of amino acids are directly oxidized to form carbonyl groups and their derivatives; the peptide chains are broken *via* both α-amidation and β-breaking; reducing sugars react with lysine to form carbonyl derivatives; oxidation with fat to form carbonyl compound conjugates (33). As shown in Figure 4B, the carbonyl content in fresh fish was low, only 0.37 nmol/g, whereas that of fish subjected to drying significantly increased, with HAD having the highest increase at 2.17 nmol/g, followed by HPD and FD (*p* < 0.05). Previous studies have shown oxidation in fish can be accelerated at higher temperatures, leading to peptide chain breakage and the oxidation of free amino and imino groups in proteins to carbonyl groups, resulting in carbonyl group accumulation (32). The higher temperature in HAD compared to other drying methods may be the reason for the greater carbonyl content observed. Protein oxidation is usually affected by ROS in the environment, while FD is carried out in vacuum and at low temperature, which isolates the effect of such radicals to a certain extent, slowing down the oxidation reaction at lower temperatures, with less carbonyl groups accumulating in FD.

A recent study suggested that protein oxidation exposes hydrophobic amino acids in the internal structure of the protein, thus increasing protein hydrophobicity to some extent (34). Surface hydrophobicity is a measure of protein denaturation through subtle changes in the physicochemical aspects of responsive protein sites and the relative content of hydrophobic amino acids on the protein surface (35). Figure 4C shows that, after drying, the surface hydrophobicity of the myofibrillar protein was elevated to some degree and significantly different from that in fresh fish (*p* < 0.05). Meanwhile, the surface hydrophobicity after FD was significantly lower than in other treatment groups. Tornberg (36) reported that temperature increases cause proteins to unfold, with protein-protein binding initiated at 36–40°C, and protein gelation occurring at 45–50°C. The gradual unfolding of the peptide chain during HAD in this study exposed the hydrophobic amino acids leading to an increase in the surface hydrophobicity of the protein. After HAD, surface hydrophobicity was insignificantly lower than observed following HPD, which might be due to certain hydrophobic residues participating in protein-protein interactions at 60°C, thus forming a network structure of aggregates and causing a decrease in hydrophobicity. Although HPD temperature was lower HAD temperature, the drying time was longer, and protein oxidation may occur under microbial activity. The surface hydrophobicity of myofibrillar proteins also increased significantly after FD. The recrystallization and sublimation of ice crystals during FD was suggested to destroy myofibrillar protein structure, thereby exposing the hydrophobic groups, which might be a reason for the increase in surface hydrophobicity (37).

### Changes in spatial conformation of myofibrillar proteins

**FIGURE 5:**
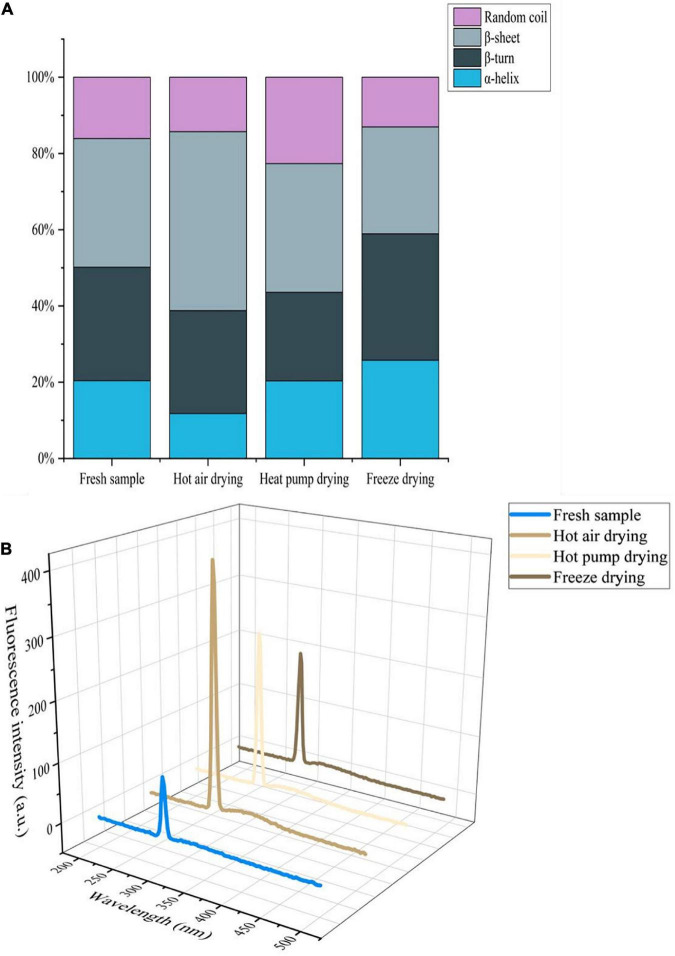
**(A)** Effects of different drying methods on the secondary structure of myofibrillar protein. **(B)** Effects of different drying methods on the endogenous fluorescence of myofibrillar protein.

In the infrared spectrum of myogenic fibronectin, the amide I band (1,600–1,700 cm^–1^) was found to be composed of C = O stretching vibrations coupled with N-H bending vibrations, which are often used to characterize secondary structural changes in myofibrillar protein owing to their high sensitivity to microenvironmental changes (38). As shown in Figure 5A, there was a significant decrease in α-helix content in myofibrillar protein after HAD, along with an increase in β-rotation angle. α-helix stability is associated with intramolecular hydrogen bonding, while protein oxidation gives rise to non-covalent interactions, including hydrogen bonding, with the α-helix undergoing deconvolution to a β-turn or β-sheet (39). Thus, high temperature compromised α-helix stability, causing α-helix deconvolution into a β-turn, with a disordered protein structure. The decrease in β-sheets and increase in that of random coils after HAD indicate that β-sheets were converted to random coils and also implies an increase in protein-protein interactions with the formation of macromolecular polymers, leading to changes in the functional properties of the myofibrillar proteins. Both α-helix and β-sheet content increased after FD, whereas the proportion of β-turns and random coils decreased. Given that moderate oxidation reportedly increases α-helix content (40), oxidation during FD may account for the observed increase in α-helix content. Moreover, myofibrillar protein structure stability is altered during freezing as a result of ice crystal formation. The increase in β-sheet content is a phenomenon of protein aggregation caused by the interaction of -SH groups forming S-S groups with hydrophobic, accompanied by an interconversion between the β-turn angle and β-sheet (41).

Aromatic amino acids such as tryptophan (Trp), tyrosine (Tyr), and phenylalanine (Phe), are located at the core of proteins; protein denaturation changes the location and microenvironment of aromatic amino acid residues. Since aromatic amino acid residues can emit fluorescence, endogenous fluorescence spectroscopy is often used to study changes in protein structure (42). As shown in Figure 5B, the fluorescence intensity of fresh fish myofibrillar protein was lowest, whereas intensity increased in the order of FD, HPD, and HAD. The maximum emission wavelength for the myofibrillar protein of fresh and FD fish was 294 nm, whereas for HAD and HPD, it was 296 and 295 nm, respectively, which reflects a red-shift compared with that of fresh fish. This indicates that the microenvironment and conformation of myofibrillar proteins were altered to a certain extent by drying. Furthermore, high-temperature treatment exposes the tryptophan side chain group of myofibrillar proteins more easily, which increases the polarity of the microenvironment and enhances the fluorescence intensity (43). In the case of HPD and FD, this may occur due to the alteration of myofibrillar protein structure caused by the effect of oxygen and water activity.

### Changes in chemical interactions

The network structure of proteins is maintained by chemical forces, including ionic bonds, hydrogen bonds, hydrophobic interactions, disulfide bonds, and non-disulfide covalent bonds, with hydrogen bonds playing an important role (44). The relative content of hydrophobic interactions was significantly increased in all dried fish compared to fresh fish (*p* < 0.05, Table 1), with the most significant increase in HAD, similar to the results of surface hydrophobicity. In addition, the relative content of ionic bonds also showed a significant decrease (*p* < 0.05) after drying, whereas that of disulfide bonds in HAD and HPD samples increased significantly. The presence of free radicals leads to the oxidation of thiol groups, which also promotes the formation of disulfide bonds; the increase in hydrophobic interactions and disulfide bonds reduces the content and mobility of immobilized water molecules, leading to a poorer fish texture. Benjakul et al. (45) suggested that disulfide bond formation induces MHC polymerization, which subsequently decreases the salt solubility of myofibrillar proteins and potentially leading to decreased ionic bonds. The relative content of hydrogen bonds was significantly increased after FD (*p* < 0.05), which might be attributed to the exposure of hydrophobic and hydrogen bonds buried in the protein under low-temperature conditions, thus altering the hydrogen bonding and hydrophobic interaction pattern (46). The main interaction in HAD and HOD fish was disulfide bonding, whereas that in fresh and FD fish it was non-disulfide covalent bonding, indicating that the protein microenvironment in fish subjected to FD was closer to that of fresh fish.

**TABLE 1:** Effect of different drying methods on chemical forces in myofibrillar protein.

Sample	Ionic bonds /%	Hydrogen bonds /%	Hydrophobic interactions /%	Disulfide bonds /%	Non-disulfide covalent bonds /%
Fresh sample	24.98 ± 2.18^a^	4.03 ± 0.36^c^	0.26 ± 0.03^d^	18.74 ± 0.27^c^	51.99 ± 2.28^a^
Hot air drying	7.19 ± 0.70^b^	4.74 ± 0.36^b^	10.01 ± 0.08^a^	68.59 ± 1.19^b^	9.48 ± 0.19^c^
Heat pump drying	4.58 ± 0.51^c^	3.41 ± 0.41^c^	3.32 ± 0.04^c^	79.73 ± 1.16^a^	8.96 ± 0.32^c^
Freeze drying	8.07 ± 0.75^b^	21.11 ± 1.80^a^	7.58 ± 0.20^b^	19.93 ± 0.64^c^	43.31 ± 1.79^b^

Values represent the mean ± SD (*n* = 3). Lower case letters indicate significance at *p* < 0.05.

### Changes in myoglobin composition

**FIGURE 6:**
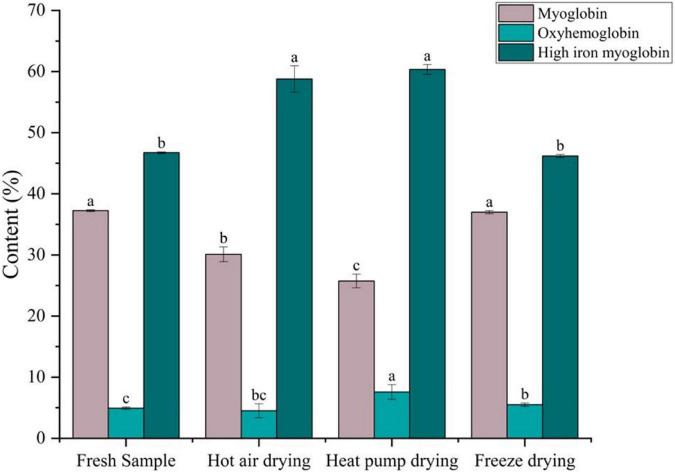
Effects of different drying methods on the content of different forms of myoglobin. Different colored bars represent different forms of myoglobin. Lower case letters indicate significance at *p* < 0.05.

Meat color is primarily determined by myoglobin, oxymyoglobin, and high-iron myoglobin, which can cause the muscle to appear dark red, bright red, and gray-brown in color, respectively (47). As shown in Figure 6, compared with fresh fish, there was no significant difference in the myoglobin or high-iron myoglobin contents between fresh and FD fish (*p* > 0.05), whereas oxygenated myoglobin content was significantly higher (*p* < 0.05), indicating that color remained unchanged after FD. After HPD, the myoglobin content decreased from 37.25 to 25.74% after HPD, whereas the high-iron myoglobin content showed a significant increase (*p* < 0.05), although the oxygenated myoglobin content was the highest. Meanwhile, high-iron myoglobin content in HPD samples was also the highest, with significant differences (*p* < 0.05), indicating that myoglobin was severely oxidized, resulting to fish samples darker in color. A comparable decrease in myoglobin content was observed after HAD, with high-iron myoglobin increasing from 46.73 to 58.78%, suggesting that browning occurred.

During drying, oxidative substances, such as ROS, accumulate in the meat. When NADH is depleted in the muscle, myoglobin and oxymyoglobin are oxidized to high-iron myoglobin in a process correlated to lipid oxidation (48). Chaijan et al. (49) reported that myoglobin can bind to myosin, and protein conjugates cause conformational changes in the globin, thus promoting the formation of high-iron myoglobin. It has been reported that high-valent myoglobin can induce protein cross-linking by reacting with myosin to generate myosin radicals (50). This may explain the lower high iron myoglobin content in HAD than in HPD samples.

## Conclusion

In this study, we investigated the effects of different drying methods on the quality of golden pompano filets. Changes in fish tissue structure after drying led to changes in texture. Analysis of the physicochemical properties revealed that both HAD and HPD samples showed significant oxidation and degradation of myofibrillar proteins, whereas FD samples showed clear protein bands and a lower degree of oxidation. In addition, the content of several forms of myoglobin indicated that the color of fish meat was better maintained after FD, whereas browning occurred after HAD and HPD.

This study demonstrated that various drying methods resulted in golden pompano products with differences in muscle tissue and protein conformation. Among the three drying methods, FD maintained tissue fiber integrity and protein structural stability to the greatest extent. Our findings provide a theoretical basis for future studies investigating quality changes in dried fish filets during processing.

## Data availability statement

The original contributions presented in this study are included in the article/supplementary material, further inquiries can be directed to the corresponding author/s.

## Author contributions

PC: writing—original draft and conceptualization. YQ: writing—original draft and data curation. SC: visualization and data curation. YZ: visualization. YYW: conceptualization. YQW: writing—review and editing. All authors contributed to the article and approved the submitted version.
